# 1-Aminocyclopropane-1-carboxylate deaminase producers associated to maize and other *Poaceae* species

**DOI:** 10.1186/s40168-018-0503-7

**Published:** 2018-06-20

**Authors:** Marie-Lara Bouffaud, Sébastien Renoud, Audrey Dubost, Yvan Moënne-Loccoz, Daniel Muller

**Affiliations:** 1 Univ Lyon, Université Claude Bernard Lyon 1, CNRS, INRA, VetAgro Sup, UMR5557 Ecologie Microbienne, F-69622 Villeurbanne, France; 2 Helmholtz Center for Environmental Research UFZ, Theodor-Lieser-Straβe 4, 06120 Halle, Germany

**Keywords:** ACC deaminase functional group, Rhizosphere community, *Poaceae* evolution

## Abstract

**Background:**

Complex plant-microbe interactions have been established throughout evolutionary time, many of them with beneficial effects on the host in terms of plant growth, nutrition, or health. Some of the corresponding modes of action involve a modulation of plant hormonal balance, such as the deamination of the ethylene precursor 1-aminocyclopropane-1-carboxylate (ACC). Despite its ecological importance, our understanding of ACC deamination is impaired by a lack of direct molecular tools. Here, we developed PCR primers to quantify the ACC deaminase gene *acdS* and its mRNA in soil communities and assessed *acdS*^+^ microorganisms colonizing maize and other *Poaceae* species.

**Results:**

Effective *acdS* primers suitable for soil microbial communities were obtained, enabling recovery of bona fida *acdS* genes and transcripts of diverse genetic backgrounds. High numbers of *acdS* genes and transcripts were evidenced in the rhizosphere of *Poaceae*, and numbers fluctuated according to plant genotype. Illumina sequencing revealed taxonomic specificities of *acdS*^+^ microorganisms according to plant host. The phylogenetic distance between *Poaceae* genotypes correlated with *acdS* transcript numbers, but not with *acdS* gene numbers or the genetic distance between *acdS* functional groups.

**Conclusion:**

The development of *acdS* primers enabled the first direct analysis of ACC deaminase functional group in soil and showed that plant ability to interact with soil-inhabiting *acdS*^+^ microorganisms could also involve particular plant traits unrelated to the evolutionary history of *Poaceae* species.

**Electronic supplementary material:**

The online version of this article (10.1186/s40168-018-0503-7) contains supplementary material, which is available to authorized users.

## Background

Growth, development, and health of macroorganisms are strongly influenced by the interactions they develop with their associated microbial community [1–3]. These interactions often involve nutrient exchanges between partners, the host typically providing organic carbon [4–7] while microorganisms supply amino acids or mineral nutrients, which results from various processes such as nitrogen fixation or phosphorus solubilization [8]. Many of these interactions are complex and entail also the exchange of molecular signals [2]. In the case of plants, microbial partners might influence host hormonal balance by producing molecules mimicking phytohormones (e.g., auxins, gibberellins, or jasmonate) or enzymes that modulate plant hormonal production, notably by degrading the ethylene biosynthetic precursor 1-aminocyclopropane-1-carboxylate (ACC) [2, 8, 9].

Ethylene is a plant hormone that regulates plant development and stress responses [10, 11]. Microorganisms able to produce ACC deaminase transform the ethylene precursor ACC into α-ketobutyrate and ammonia [12, 13]. By degrading ACC within roots or in exudates (thereby leading to a sink effect), root-interacting bacteria are indirectly lowering ethylene level in plant roots, thus stimulating root growth and modulating plant stress resistance [9, 14–17]. The *acdS* gene encoding ACC deaminase is highly conserved among microorganisms and has been used to study the phylogeny and diversity of ACC deaminase producers [18–20] in bacteria and micro-eukaryotes (i.e., fungi and stramenopiles). Although horizontal transfer of *acdS* between bacteria was suspected due to incongruence between *acdS* and 16S rRNA gene-based bacterial phylogenies [21, 22], a more exhaustive assessment suggested that *acdS* was mainly inherited vertically, with only occasional horizontal gene transfers [20]. Thus, *acdS* is a marker suitable to assess complex ACC deaminase functional communities.

In the root zone, the plant may be colonized by different types of *acdS*^*+*^ microorganisms [23, 24], which are likely to contribute jointly to degradation of ACC produced by roots, and the overall significance of ACC deamination for the plant is expected to result from the combined functioning of its *acdS*^*+*^ microbial partners [2]. However, even though various kinds of *acdS*^*+*^ microorganisms can be readily isolated from the rhizosphere, there is no direct PCR tool available to assess the entire functional group of root-associated *acdS*^*+*^ microorganisms, i.e., including non-cultured taxa and strains.

It is long established that plants shape their rhizosphere bacterial community from the telluric bacterial reservoir [25, 26]. Plant species and plant genotypes within species exhibit specific phenotypic traits, including root properties that are likely to influence rhizobacterial community composition [7]. And indeed, several studies showed the impact of plant genotype on the taxonomic composition of their associated bacterial community [27–30]. This type of effect has also been evidenced when assessing the genetic diversity of functional groups important for plant growth, such as 2,4-diacetylphloroglucinol-producing pseudomonads [31–33], nitrogen fixers [34], or microorganisms involved in other biogeochemical transformations [35], and it might well be that similar effects also take place with *acdS*^*+*^ microorganisms.

Therefore, this work aimed at testing the hypothesis that the functional group of *acdS*^*+*^ microorganisms differed according to plant genotype, which was done in the case of *Poaceae*. To this end, protocols for quantitative PCR (qPCR) and quantitative reverse-transcription PCR (qRT-PCR) of *acdS* were developed and validated for analysis of *acdS*^*+*^ microorganisms within the rhizosphere. These tools as well as *acdS* MiSeq sequencing were implemented on eight *Poaceae* genotypes previously used in Bouffaud et al. [36] and enabling comparisons at different *Poaceae* taxonomic levels, i.e., between individual inbred lines within two maize genetic groups (Corn Belt Dent and Northern Flint), between two maize genetic groups, between these maize genetic groups and a teosinte (representing maize’s pre-domestication *Zea mays* ancestor), and between *Z. mays* and another member (sorghum) from maize’s *Panicoideae* subfamily or a member (wheat) from the neighboring *Pooideae* subfamily.

## Results

### Validation of qPCR and qRT-PCR tools

We developed primer sets (Additional file 1: Table S1) to specifically amplify *acdS* gene sequences in the *acdS* reference database constructed. Eight primer pairs were discarded as they failed to specifically amplify *acdS* from pure bacterial genomic samples. Another one was discarded as it could not amplify *acdS* genes from a soil community, and only pair acdSF5/acdSR8 (Additional file 1: Tables S1, S2 and Additional file 2: Figure S1) was kept.

qPCR conditions were optimized to obtain an amplification efficiency of > 80% and an error below 0.1 with genomic DNA of *acdS* strains *P. kilonenesis* F113 (i.e., 81.2% and 0.06, respectively) and *Burkholderia cenocepacia* J2315 (i.e., 100% and 0.007, respectively). The detection limit of the qPCR method on pure cultures was 6 *acdS* gene copies (corresponding to 50 fg of J2315 genomic DNA template**)**. When tested on total DNA or cDNA (reverse transcription on total RNA) obtained from rhizosphere of the different *Poaceae*, amplification efficiencies above 80% and errors below 0.1 were also obtained, for both soils tested. *acdS* could always be detected by qPCR in the two bulk soils (Additional file 3: Figure S2).

Illumina MiSeq sequencing of acdSF5/acdSR8 amplicons from bulk soils and rhizosphere soils gave 3,903,982 reads (44,287 OTUs) from cropped soil and 1,673,758 reads (28,759 OTUs) from meadow soil. Similarities of *acdS* sequences with known references were assessed with the in-house core-*acdS* database extracted from the FunGene database. The phylogenetic tree showed that none of the sequences clustered within the D-cystein-sulfydrase outgroup (Additional file 4: Figure S3). The OTUs obtained clustered in numerous clades encompassing all the known diversity of the *acdS* gene (*Proteobacteria*, *Actinobacteria*, and microeukaryotes; Additional file 4: Figure S3).

### Size of *acdS* group and number of *acdS* transcripts in *Poaceae* rhizosphere

**Fig. 1:**
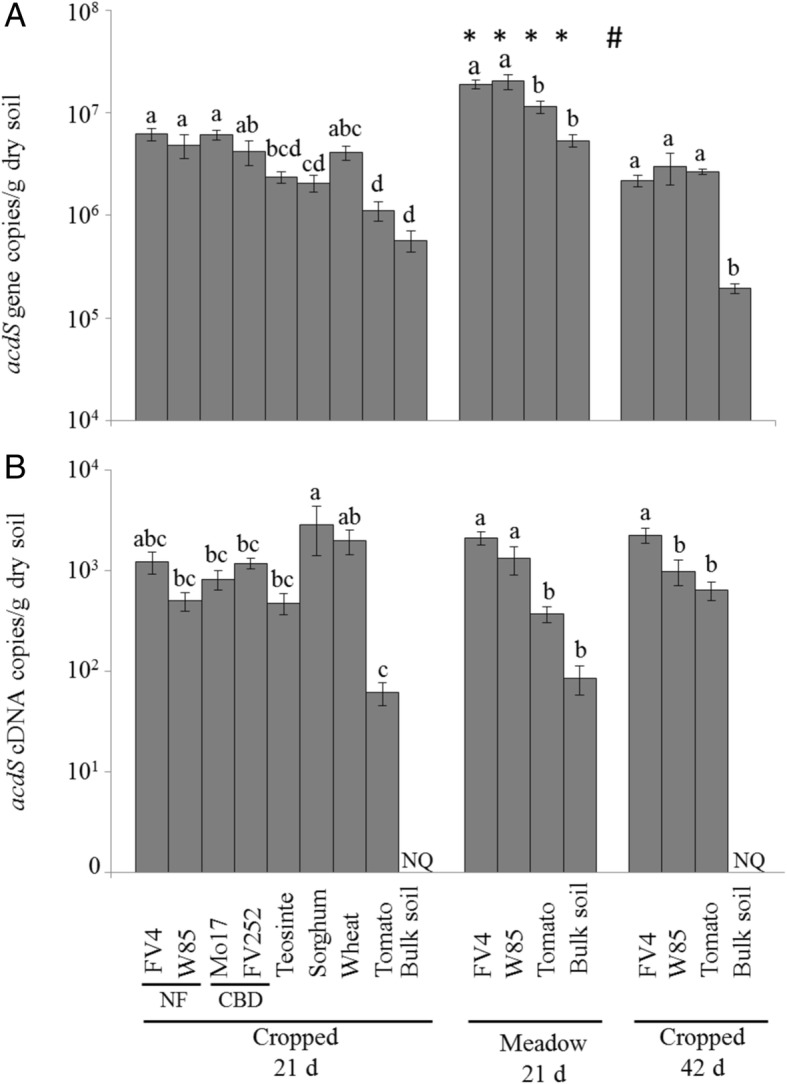
Quantification of *acdS* genes (**A**) and *acdS* transcripts (**B**) in bulk soil and rhizosphere of *Poaceae* genotypes and tomato by qPCR and qRT-PCR, respectively. NF means Northern Flint and CBD, Corn Belt Dent. NQ: not quantifiable. Statistical analyses were performed independently at 21 days in cropped soil, at 21 days in meadow soil, and at 42 days in cropped soil, using ANOVA and Fisher LSD tests (*P* < 0.05; results shown with letters a to d). For FV4, W85, tomato, and bulk soil, two-way ANOVA and Fisher LSD tests (*P* < 0.05) were also performed to compare treatments according to past soil management or sampling time, and differences with the same genotype at 21 days in cropped soil are indicated by symbols * and #, respectively

The size of the *acdS* group amounted to 0.5–6.2 × 10^6^ *acdS* gene copies per g of rhizosphere soil in the cropped soil at 21 days, 0.5–2.0 × 10^7^ *acdS* copies in the meadow soil at 21 days, and 0.2–3.0 × 10^6^ *acdS* copies in the cropped soil at 42 days (Fig. 1A). Compared to bulk soil, *acdS* group size was higher in the presence of plant for all four maize lines and wheat at 21 days in cropped soil (all five displaying similar levels), for the two maize lines in the meadow soil at 21 days, and for all plants in the cropped soil at 42 days. A significant effect of past soil management was observed in all four treatments studied (two maize lines, tomato, and bulk soil), but an influence of sampling time was evidenced only for maize line FV4.

The number of *acdS* transcripts reached 0.06–2.9 × 10^3^ *acdS* cDNA copies per g of rhizosphere soil in the cropped soil at 21 days, 0.08–2.1 × 10^3^ *acdS* copies in the meadow soil at 21 days, and 0.6–2.2 × 10^3^ *acdS* copies in the cropped soil at 42 days (Fig. 1B). The *acdS* transcripts in the bulk soil samples from cropped soil at 21 and 42 days were below the quantification limit. A strong positive influence of *Poaceae* on the number of *acdS* transcripts was observed in both soils at both sampling times. In addition, *acdS* transcript number in cropped soil was higher for sorghum than maize (except FV4) and teosinte (and tomato) at 21 days, and for FV4 maize than W85 (and tomato) at 42 days, while it was higher for both maize lines studied than for tomato and bulk soil in meadow soil at 21 days. Overall, no significant effect of past soil management or sampling time was found for *acdS* transcript number.

### Diversity of *acdS*^+^ microorganisms colonizing *Poaceae*

Rarefaction analyses resulted in different saturation profiles, suggesting that *acdS* allele diversities differed across the different *Poaceae* rhizospheres (Additional file 5: Figure S4). To estimate *acdS* richness, a subsampling was done with 38,524 sequences per cropped soil sample and with 25,423 sequences per meadow soil sample.

Shannon, Simpson, and Chao diversity indices were calculated for each sample (Tables 1 and 2). In the cropped soil, no significant difference in the diversity of the *acdS* functional group was observed between treatments. In the meadow soil, Shannon (*P* = 0.038) and Simpson (*P* = 0.044) indices differed between FV4 maize and tomato (but showed opposite trends), whereas the difference was not significant for the Chao index.

**Table 1:** Diversity indices of *acdS* functional group. Shannon, Simpson, and Chao indices in bulk soil and rhizosphere were determined at 21 days of plant growth in cropped soil

	Bulk soil	NF maize FV4	NF maize W85	CBD maize Mo17	CBD maize FV252	Teosinte	Sorghum	Wheat
Shannon	7.12 ± 0.33	6.26 ± 1.63	7.54 ± 0.19	7.18 ± 0.70	6.93 ± 0.14	7.21 ± 0.41	7.23 ± 0.11	7.28 ± 0.16
Simpson	0.0051 ± 0.0039	0.0613 ± 0.1174	0.0028 ± 0.0009	0.0075 ± 0.0102	0.0076 ± 0.0044	0.0076 ± 0.0044	0.0030 ± 0.0006	0.0054 ± 0.0041
Chao	14,326 ± 2006	12,578 ± 2984	15,996 ± 604	14,156 ± 3100	13,783 ± 579	14,030 ± 3095	14,049 ± 439	15,585 ± 1237

*NF* Northern Flint genetic group of maize, *CBD* Corn Belt Dent genetic group of maize. Data are means ± standard deviations (*n* = 5)

**Table 2:** Diversity indices of *acdS* functional group. Shannon, Simpson, and Chao indices in bulk soil and rhizosphere were determined at 21 days of plant growth in meadow soil

	Bulk soil	NF maize FV4	NF maize W85	Tomato
Shannon	6.09 ± 1.26 ab	6.71 ± 0.16 a	6.68 ± 0.13 ab	6.19 ± 0.25 b
Simpson	0.054 ± 0.089 ab	0.009 ± 0.001 a	0.010 ± 0.002 ab	0.020 ± 0.007 b
Chao	12,870 ± 1882	12,353 ± 730	12,814 ± 834	11,957 ± 1316

*NF* Northern Flint genetic group of maize, *CBD* Corn Belt Dent genetic group of maize. Data are means ± standard deviations (*n* = 5). Statistical differences (when any) are indicated using letters a and b (Kruskal-Wallis tests; *P* < 0.05)

**Fig. 2:**
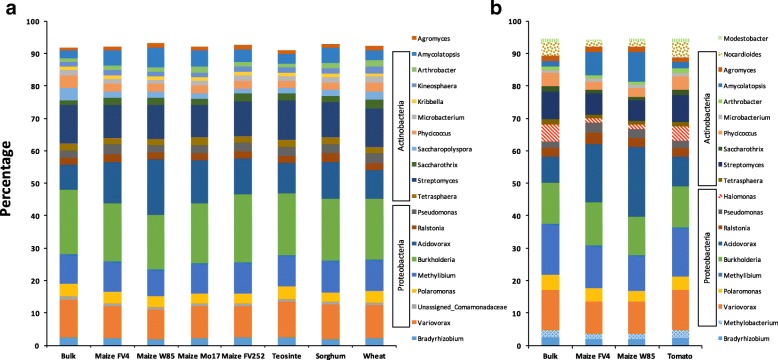
Relative abundance of the 20 most abundant *acdS* bacterial genera (all *Proteobacteria* or *Actinobacteria*) present in bulk soil and common to each rhizophere at 21 days in cropped (**a**) or meadow (**b**) soil. The remaining sequences (< 8% in cropped soil and < 6% in meadow soil) are not pictured. Mean values across six replicates are shown for each treatment. The same colors were used in **a** and **b** for genera common to both soils

When considering taxa corresponding to the *acdS* alleles obtained, the 20 most abundant genera thus identified (all bacterial) were common to all samples and represented over 90% of the sequences (Fig. 2). Compared to bulk cropped soil, the relative abundance of the 20 most abundant taxa in the *acdS* community was largely similar in the rhizosphere of the different Poaceae. However, the genus *Saccharotrix* (1.44 ± 0.18% in bulk soil) reached as much as from 2.09 ± 0.09% (maize W85) to 2.64 ± 0.37% (maize FV252) in the rhizosphere, *Amycolatopsis* (2.49 ± 0.15% in bulk soil) from 2.90 ± 0.20% (wheat) to 6.14 ± 1.78% (maize W85), and *Acidovorax* (7.59 ± 0.67% in bulk soil) from 8.71 ± 0.27% (wheat) to 17.09 ± 0.15% (maize W85). In parallel, the genus *Saccharopolyspora* (3.77 ± 0.76% in bulk soil) represented only from 2.56 ± 0.54% (wheat) to 1.21 ± 0.30% (maize FV252) of all *acdS* sequences in the rhizosphere, and *Phycicoccus* (3.99 ± 0.70% in bulk soil) from 2.76 ± 0.56% (wheat) to 2.20 ± 0.22% (sorghum). Similar dynamics were observed in meadow soil when comparing bulk soil with two maize lines (FV4 and W85 from the same maize genetic group) and the non-*Poaceae* reference tomato (Fig. 2).

**Fig. 3:**
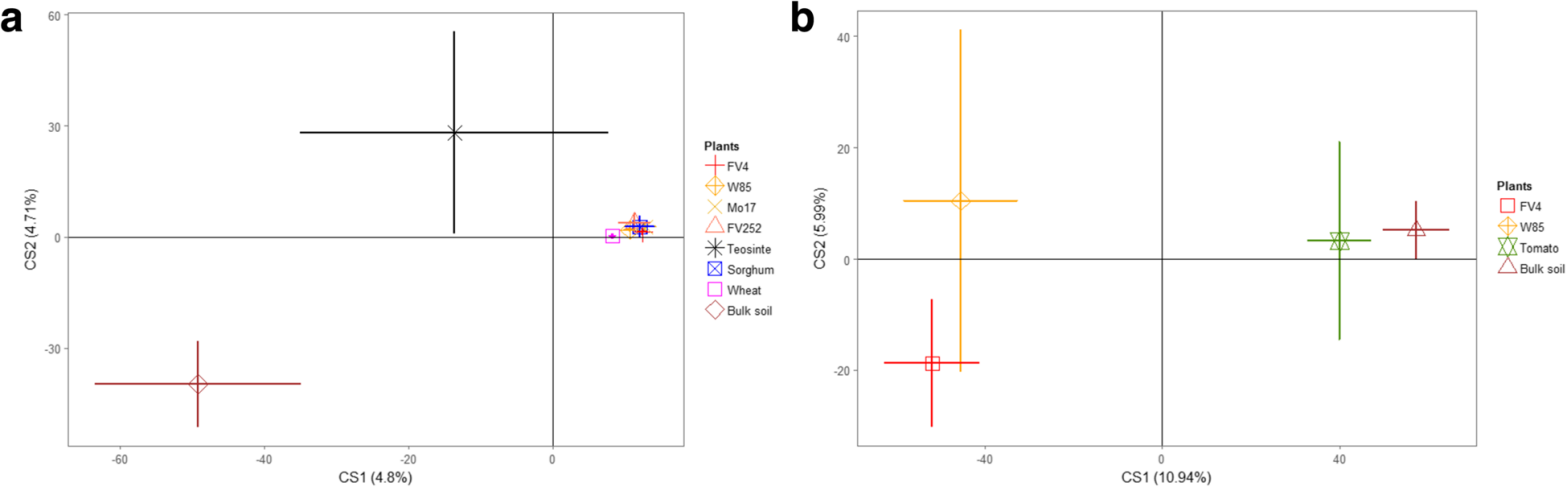
Between class analysis for comparison of *acdS* functional group diversity in bulk soil and the rhizosphere of different *Poaceae* grown in cropped-soil (**a**) or of maize and tomato in meadow soil (**b**). FV4 and W85 are Northern Flint and Mo17 and FV252 are Corn Belt Dent. For each treatment, the mean and standard error computed using all data are presented (*n* = 6)

For a more global appraisal, *acdS* sequence data were also processed by between-class analysis, which showed that *acdS* community composition in the rhizosphere differed from that in bulk soil at 21 days in cropped soil (Fig. 3a). In addition, a difference was also found between cultivated (maize, sorghum, and wheat) and spontaneous *Poaceae* (teosinte) (Fig. 3a). A significant rhizosphere effect also took place in the meadow soil, but it was of modest magnitude for the non-*Poaceae* reference tomato (Fig. 3b).

### Relationship between *Poaceae* evolution and *acdS* functional group

**Fig. 4:**
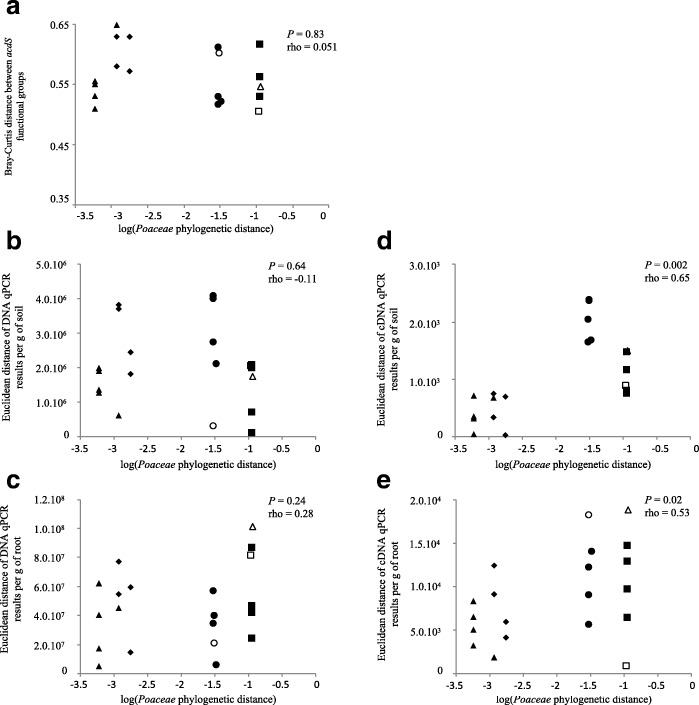
Pairwise comparison of plant phylogenetic distance between *Poaceae* (*X*-axis) with the corresponding distance between their root-associated ACC deaminase functional group (*Y*-axis). The 18 maize-based comparisons are indicated using black triangles (maize-maize; *n* = 6), diamonds (maize-teosinte; *n* = 4), circles (maize-sorghum; *n* = 4), or squares (maize-wheat; *n* = 4), and the three other comparisons using a white circle (teosinte-sorghum), white triangle (teosinte-wheat), or white square (sorghum-wheat). Distances were calculated two by two, using Kimura two-parameter model for plant phylogeny, Bray-Curtis distance between rhizobacterial communities (**a**) and Euclidean distance for qPCR (**b**, **c**) or qRT-PCR data (**d**, **e**). The Spearman correlation coefficient is indicated

The assessment of the influence of past *Poaceae* evolution on recruitment of *acdS* microorganisms did not yield any significant correlation, regardless of whether pairwise *Poaceae* phylogenetic distances were crossed with (i) differences in raw numbers (or log-numbers; Additional file 1: Table S3) of *acdS* genes copies per g of rhizosphere soil (*P* = 0.64; Fig. 4b) or g of root (*P* = 0.24; Fig. 4c), or (ii) differences in pairwise Bray-Curtis dissimilarity indices of *acdS* communities (*P* = 0.83; Fig. 4a), at 21 days in the rhizosphere of *Poaceae* grown in cropped soil. When considering the functioning of the *acdS* community, however, a significant correlation with pairwise *Poaceae* phylogenetic distances was found when computing differences in raw numbers (but not log numbers; Additional file 1: Table S3) of *acdS* transcript copies per g of rhizosphere soil (Spearman rho = 0.65; *P* = 0.002; Fig. 4d) or g of root (Spearman rho = 0.53; *P* = 0.02; Fig. 4e).

### Relationship between maize/*Poaceae* evolution and particular rhizobacterial populations

Among the 20 most abundant genera representing over 90% of *acdS* functional group diversity, a significant positive correlation (*P* < 0.05) was found for four *Actinobacteria*, i.e., *Agromyces* (rho = 0.61; *P =* 0.006), *Kineosphera* (rho = 0.61; *P =* 0.005), *Saccharopolyspora* (rho = 0.57; *P =* 0.009), and *Phycicoccus* (rho = 0.51; *P =* 0.022) when comparing read abundance for these genera to the log value of the maximum likelihood phylogenetic distance between *Poaceae* (Additional file 6: Figure S5).

## Discussion

Since the rhizosphere microbiota results mostly from the selection of soil-residing microorganisms [25, 26], root exudates and other rhizodeposits are expected to play a major role [5], which could explain why this microbial selection can differ according to host genetics [37]. Indeed, plant genetic background was shown to be an important factor shaping the root-associated microbiota, when considering both taxonomic [28, 29, 36, 38] and functional microbial groups, e.g., 2,4-diacetylphloroglucinol-producing pseudomonads [39] or nitrogen-fixing bacteria [34].

In the last two decades, much has been done to document the impact of plant genotype and/or development stage on rhizosphere microbial diversity, with an emphasis on taxonomic assessments using 16S and 18S rRNA genes as well as internal transcribed spacers [40]. However, these taxonomic assessments do not tell much about the functional potential of the microbiota, and this gap limits our capacity to understand the functioning of the plant holobiont [41]. This issue may be targeted at the scale of the entire microbial community, by metagenomics or metatranscriptomics [28, 42], as well as with a focus on particular ecological functions of prime significance, such as nitrogen fixation [34, 43, 44], nitrification [44, 45], or production of antimicrobial metabolites [46].

For certain key functional groups of soil microorganisms, primers and PCR protocols necessary for direct analysis of soil and rhizosphere populations have been used for years (e.g., [33, 46, 47]), but for others, the tools are not available yet. This was the case in particular for ACC deaminase-positive microorganisms, despite the research emphasis put on this microbial function in the last 20 years (> 10,000 papers on these microorganisms as listed in Google Scholar). The ecological importance of microbial ACC deaminase activity derives from its effect on plant metabolism of ethylene, a major phytohormone involved in various plant physiological pathways such as root elongation and immune response [16]. ACC itself acts as a phytohormone [17]. Root-colonizing microorganisms with ACC deaminase activity have the potential to indirectly lower endogenous plant levels in ACC and ethylene, and thus to alleviate environmental stress in plant and enhance root growth [16, 48].

Against this background, our first objective was to develop an *acdS*-based qPCR method targeting all *acdS* alleles, which was successfully achieved by using a set of *acdS* strains representative of the highest diversity possible of documented alleles (from *Azospirillum lipoferum* CRT1 to *Ralstonia solanacearum* GMI100; Additional file 4: Figure S3). This is first indicated by the ability of the method to minimize *acdS* quantification bias, as qPCR performance was satisfactory for two contrasted *acdS* strains based on the criteria of Zhang and Fang [49] (i.e., *R*^2^ > 0.95 and slope between − 3.0 and − 3.9 corresponding to PCR efficiencies of 80–115%). Second, it is also indicated by (i) primer specificity and (ii) the possibility to target the known diversity of *acdS* alleles in *Proteobacteria*, *Actinobacteria*, *Deinococcus/Thermus*, and micro-eukaryotes [19, 20, 50], as revealed by MiSeq Illumina sequencing (Additional file 4: Figure S3). Apart from the absence of sequences affiliated to the thermophilic genus *Meiothermus* of the *Deinococcus/Thermus* phylum (the unique genus of this phylum shown to harbor *acdS* [20]), all the other clades were represented, without over-representation of a given type of *acdS* sequence (Fig. 2). Therefore, this work proposes a novel tool that can be used to monitor the size, transcription, and diversity of the *acdS* functional group in various plant, soil, and environmental conditions.

Our second and main objective was to assess the influence of past *Poaceae* evolution on root interactions with *acdS* microorganisms, based on the rationale that bacterial taxa harboring *acdS* strains colonize roots of different *Poaceae* [27, 28, 30], *acdS* microorganisms are readily isolated from these plants [2, 21, 51, 52], and they can stimulate growth of cereals [53–56]. Recruitment of *acdS* microorganisms by all *Poaceae* was indeed shown by qPCR (Fig. 1), and it might be an indication of the importance of ACC deamination for the plant. Accordingly, we found a strong increase in the numbers of *acdS* ARNm (cDNA) when comparing the rhizosphere to bulk soil, showing for the first time the stimulation of *acdS* group functioning on roots. It can be expected that microbial functioning varies with different plant species [57, 58], and in this work, *acdS* transcript levels in the rhizosphere differed according to *Poaceae* genotype. Although rhizosphere effects were not the same for all *Poaceae* genotypes, past *Poaceae* evolution did not appear to have played a role on root selection of *acdS* microorganisms, as correlations were not significant when considering the size or genetic make-up of the *acdS* functional group in the rhizosphere. In fact, when considering the relationship obtained by Bouffaud et al. [36] between (i) the phylogenetic distance between *Poaceae* genotypes and the genetic distance between rhizobacterial communities, *Proteobacteria* and *Actinobacteria* (the main bacterial phyla containing *acdS* microorganisms) contributed strongly to the relationship but this concerned only 4 of the 20 principal proteobacterial and actinobacterial taxa evidenced in the current work, i.e., *Pseudomonas*, *Acidovorax*, *Burkholderia*, and *Variovorax*. Furthermore, we found that none of these four proteobacterial genera showed any correlation with Poaceae evolutionary history, whereas four actinobacterial genera not characterized in Bouffaud et al. [36] did show a significant correlation. This is reminiscent of previous findings with the functional group of diazotrophic bacteria [28, 34], and it suggests that selection for particular types of function-providing microorganisms may proceed following somewhat different ecological rules than those that apply to all members of the corresponding taxa [41].

## Conclusion

In summary, we report the first *acdS*-based PCR method to monitor *acdS* alleles and transcripts of the ACC deaminase functional group in natural soil and plant systems. We then analyzed the microbial ACC deaminase functional group in the context of *Poaceae* evolutionary history. Correlations were significant when considering pairwise differences in raw numbers of *acdS* transcripts versus the genetic distance between *acdS* groups. This is the first evidence of a link between past *Poaceae* evolution and the functioning of a root-associated microbial group, such a link having not been observed for instance when considering N-fixing bacteria [34]. It also raises the possibility that microbial modulation of ethylene metabolism has evolved to different extents in various *Poaceae* lineages, and this issue will deserve further research attention.

## Methods

### Identification of *acdS* primers

All available (in February 2016) sequences from the *acdS*/D-cysteine-sulfhydrase FunGene sequence database were retrieved and aligned using MUSCLE. Key amino acid positions known to be important for ACC deaminase activity, namely Lys51, Ser78, Tyr295, Glu296, and Leu322 [59] were searched using *Pseudomonas kilonensis* F113 as a reference. Sequences presenting different amino acids in the key positions were discarded, as they are likely to correspond to D-cysteine desulfhydrases [59].

The *acdS* sequences and *acdS* homologs (also included in the alignment to serve as non-target sequences) from the D-cysteine desulfhydrase producer strains *Pseudomonas syringae* DC3000 (AE16853), *Achromobacter xylosoxidans* BM1 (AY604540), *Achromobacter* sp. CM1 (AY604541), *Rhizobium* sp. TAL1145 (EU183544), *Serratia proteamaculans* SUD165 (AY604543), *Escherichia coli* K12 (CP014348), *Pseudomonas marginalis* (AY604542), and *Enterobacter aerogenes* Cal3 (AY604544) were aligned using MUSCLE [60]. This alignment was used to perform a phylogenetic analysis on *acdS* sequences. The tree was inferred from 1000 nucleotides using the neighbor-joining (NJ) method in MEGA4 [61] with the Kimura two-parameter method for distance calculation [62]. Nodal robustness of the tree was assessed using 1000 bootstrap replicates.

Using the *acdS* alignment, primers (25–30 bp in length) were visually selected in regions conserved among the *acdS* sequences and absent from the non-target sequences. The new primers (Additional file 1: Table S1) were then assessed based on the following criteria: (i) a melting temperature (Tm) of 60 to 67 °C, (ii) an absence of predicted hairpin loops and primer-dimer formations [63], (iii) a Tm difference between primers not exceeding 1 °C, (iv) an amplification product not exceeding 300 bp, (v) a maximum of three mismatches between each primer and the 1304 *acdS* sequences, and (vi) the ability to specifically amplify *acdS* in genomic DNA samples (using 6 ng of genomic DNA from the *acdS*^*+*^ strains *Azospirillum lipoferum* TVV3, 4B and RSWT1, *Burkholderia cepacia* LMG 1222, *B. cenocepacia* LMG 16656 and J2315, *Burkholderia stabilis* LMG 14294, *Burkholderia dolosa* LMG18941, *Pseudomonas thivervalensis* PITR2, *P. kilonensis* F113, *Ralstonia solanacearum* GMI1000, and the non-*acdS* strains *Pseudomonas protegens* CHA0, *E. coli* K12, and *A. lipoferum* CRT1). Primer melting temperature, predicted hairpin loops, and predicted primer-dimer formations were determined using Oligo 6 (Molecular Biology Insights, West Cascade, CO) and the nearest-neighbor method [64]. Amplification specificity was determined by checking the Tm and size of the amplification product through (i) melting curve analysis followed by Tm determination (described below) and (ii) gel electrophoresis analysis and the observation of a single band of the expected size. Following this, one *acdS*-specific primer pair was selected for development of *acdS* qPCR and qRT-PCR protocols.

### Development of *acdS* qPCR and qRT-PCR

Quantitative PCR assays were conducted using LightCycler 480 SYBR Green I Master mix in a final volume of 20 μL and a LightCycler 480 (Roche Applied Science, Meylan, France). Cycle threshold (Ct) of individual samples was calculated using the second derivative maximum method in the LightCycler Software v.1.5 (Roche Applied Science). The standard curves were obtained by plotting the mean Ct value of the three replicates (per DNA concentration) against the log-transformed DNA concentration. Amplification efficiency (E), calculated as *E* = 10^(−1/slope)^ − 1, and the error of the method (mean squared error of the standard curve) were determined using the LightCycler Software v.1.5 (Roche Applied Science). Standard curves were generated using genomic DNA of *P. kilonensis* F113 and *B. cenocepacia* J2315, two bacterial genomes harboring a single *acdS* copy. *acdS* copy number was computed as [DNA (g) × Avogadro’s number (molecules mol^−1^)]/[number of DNA base pairs in *acdS* fragment ×  660 (g mol^−1^)], based on an average of 660 g mol^− 1^ per base pair. Amplification specificity was assessed by melting curve analysis of PCR products, done by ramping the temperature to 95 °C for 10 s and back to 65 °C for 15 s, followed by increases of 0.1 °C s^−1^ up to 95 °C.

qPCR optimization was sought to improve *acdS* amplification efficiency (above 80%) and error (below 0.01) for the *acdS* strains *P. kilonensis* F113 and *B. cenocepacia* J2315. Three primer concentrations (0.5, 0.75, and 1 μM), four annealing temperatures (66, 67, 68, and 70 °C), two annealing times (30 and 15 s), and three elongation times (30, 15, and 10 s) were tested.

The final qPCR protocol used primers acdSF5/acdSR8 amplifying a fragment of 133 nt. Reaction mix contained 10 μL of LightCycler 480 SYBR Green I Master (Roche Applied Science), 1 μM of each primer, and 2 μL of DNA extract. The final cycling program included a 10-min incubation at 95 °C, 50 amplification cycles of 30 s at 94 °C, 7 s at 67 °C, and 15 s at 72 °C, and the fusion program for melting curve analysis described above. The generated standard curve from genomic DNA of *B. cenocepacia* J2315 was subsequently used as the external standard curve for determination of *acdS* copy number in uncharacterized DNA samples. Two DNA standards from genomic DNA of *B. cenocepacia* J2315 were included as reference in each run to detect between-run variations.

### Greenhouse experiment

We used samples from a previous greenhouse experiment [36] performed with one wheat (*Triticum aestivum* L. cv. Fiorina; Agroscope, Changins, Switzerland), one sorghum (*Sorghum bicolor* L. cv. Arprim; Semences de Provence, Fourques, France), one teosinte (*Zea mays ssp. parviglumis*; UNAM, Cuernavaca, Mexico), four maize (*Zea mays* L.) inbred lines (FV252 and Mo17 from group Corn Belt Dent, and FV4 and W85 from group Northern Flint; INRA, St Martin de Hinx, France), and one tomato (*Solanum lycopersicum* L. cv. Marmande; Vilmorin, La Ménitré, France) considered as an arbitrary, external (non-Poaceae) reference.

The plants were cultivated by Bouffaud et al. [36] in one or two sieved (6 mm) soils collected from the topsoil of two neighboring fields (luvisols) at La Côte Saint-André (France). All were grown in soil from a maize-monocropping field (loam, organic matter 2.3%, pH_H20_ 7.3, N 1.6 g kg^−1^) and three selected genotypes (see below) also in soil from a permanent meadow (loam, organic matter 5.5%, pH_H20_ 6.0, N 3.2 g kg^− 1^). In short, pots of 3 dm^3^ holding 2.5 kg soil were sown with surface-disinfected seeds (to get one seedling per pot) or kept as non-planted pots, with five pots per treatment, and they were placed in a greenhouse (randomized blocks). For all treatments, samples were taken at 21 days in cropped soil, whereas maize lines FV4 and W85 and tomato were also studied at 42 days in the same soil and at 21 days in meadow soil. Root systems were individually unearthed and shaken to discard loosely adhering soil. Roots and tightly adhering soil were frozen in liquid nitrogen and lyophilized, and rhizosphere soil was collected from roots (0.5–6 g soil per plant) and placed at − 20 °C. Root, rhizosphere soil, and shoot dry weights were measured (Additional file 7: Figure S6). Bulk soil was taken from each non-planted pot (5 g) at 21 (two soils) and 42 days (cropped soil), frozen, lyophilized, and stored at − 20 °C.

### Extraction of DNA and RNA from rhizosphere and bulk soil

We used total nucleic acids that were extracted by Bouffaud et al. [36], as follows. Briefly, 0.5 g bulk or rhizosphere soil, 0.5 ml extraction buffer (5% hexadecyltrimethylammonium bromide, 1 mM 1,4-dithio-DL-threitol, in a 0.12 M phosphate buffer at pH 8), and 0.5 g zirconium beads (VWR, Fontenay-sous-Bois, France) were used in a bead beater (TissueLyser II Retsch; Qiagen, Courtaboeuf, France) for 90 s at 30 m s^−1^. After 10 min centrifugation at 16,000*g*, supernatants were extracted twice in phenol-chloroform-isoamyl alcohol (24:24:1 *v*/*v*/*v*) and once in chloroform-isoamyl alcohol (24:1 *v*/*v*). Nucleic acids were precipitated overnight using absolute ethanol and potassium acetate (3 M, pH 4.8) at − 20 °C. After centrifugation for 30 min at 16,000*g*, pellets were washed in 70% ethanol and dissolved in 100 μL RNase-free DNase-free water (giving 50–100 ng nucleic acids μL^−1^).

### Reverse-transcription synthesis of cDNA

DNA-free RNA was obtained by treating 20 μL of nucleic acid solution with 4 U of DNase I (Invitrogen, Cergy Pontoise, France) in 1× DNase I reaction buffer at room temperature. DNA digestion repeated to remove remaining DNA traces, the reaction was stopped in presence of 1 μL of 25 mM EDTA (10 min at 65 °C), and RNA was purified with RNeasy Mini kit (Qiagen) according to manufacturer’s protocol. DNA contamination after the DNase I treatment of RNA samples was indicated by lack of qPCR amplification (performed as described below), and in the few cases where amplification did take place, an additional DNase I treatment was performed and no qPCR amplification took place then.

Total cDNA synthesis was carried out with 8 μL of resulting purified RNA extract, using random hexanucleotide primers (Invitrogen) and Omniscript reverse transcription kit (Qiagen) following the manufacturer’s instructions (90 min at 37 °C). The reverse transcriptase was inactivated 10 min at 95 °C, and cDNA was stored at − 20 °C.

### qPCR and qRT-PCR analyses of *acdS*

The quantities of *acdS* genes (qPCR) and mRNA (qRT-PCR) were estimated using 20-μL containing 4 μL of PCR grade water, 1 μL of each primer (final concentration 1 μM), 10 μL of LightCycler-DNA Master SYBR Green I master mix (Roche Applied Science), and 2 μL of sample DNA (10 ng). The cycling program entailed 10 min incubation at 95 °C, followed by 50 cycles of 94 °C for 15 s, 67 °C for 15 s, and 72 °C for 10 s. The fusion program for melting curve analysis is described above. Amplification specificity was assessed by melting curve analysis of PCR and RT-PCR products, done by ramping the temperature to 95 °C for 10 s and back to 65 °C for 15 s, followed by increases of 0.1 °C s^−1^ up to 95 °C. Melting curve calculation and determination of Tm values were performed using the polynomial algorithm function of LightCycler Software v.1 (Roche Applied Science).

Standard curves were obtained using DNA from *B. cenocepacia* J2315, whose genome contains one *acdS* copy, after diluting in triplicate from 5 × 10^−9^ to 5 × 10^−15^ g DNA μL^−1^. PCR efficiency was derived from standard curves as *E* = 10^(−1/slope)^. All five samples for rhizosphere or bulk soil treatment were assessed, and data expressed in g μL^−1^ were converted into numbers of *acdS* copies computed as [DNA (g) × Avogadro’s number (molecules mol^− 1^)]/[number of DNA base pairs in PCR template ×  660 (g mol^−1^)], based on an average of 660 g mol^−1^ per base pair.

### *acdS* sequencing from rhizosphere DNA

Primer specificity was assessed by Illumina MiSeq sequencing of acdSF5/acdSR8 amplicons (size ~ 133 nt), using bulk soils and rhizosphere soils from tomato and six *Poaceae* cultivated in cropped or meadow soil. DNA extracts were sent to MR DNA laboratory (www.mrdnalab.com; Shallowater, TX) for *acdS* sequencing. The qPCR primers acdSF5/acdSR8 were used for the sequencing library, the forward primer carrying a barcode. The 30-cycle PCR (done five times, hence 150 cycles in total) used the HotStarTaq Plus Master Mix Kit (Qiagen, Valencia, CA) with 94 °C for 3 min, followed by 28 cycles of 94 °C for 30 s, 53 °C for 40 s, and 72 °C for 1 min, and a final elongation at 72 °C for 5 min. Samples were pooled together in same proportions, purified with calibrated Ampure XP beads and the DNA library was obtained with Illumina TruSeq DNA library protocol. Sequencing was carried out on a MiSeq following the manufacturer’s instructions.

Sequence data were treated with MR DNA pipeline. Briefly, sequences were depleted of barcodes, sequences < 120 bp or > 160 bp or with ambiguous base calls removed, the remaining sequences denoised, operational taxonomic units (OTUs; defined at 3% divergence threshold) generated, and chimeras removed. OTUs were then classified using BLASTn and a curated *acdS* database (described in *acdS* database supplemental material). Briefly, the *acdS* in-house database (see the “Identification of acdS primers” section) developed to define the qPCR primers was adapted to exhibit only the 133 nt corresponding to the amplified PCR fragments. Moreover, when different accessions presented 100% identity in nucleotide sequences and were affiliated to a same bacterial species, only one sequence was kept, reducing the database entries to 1304 different sequences (named core-*acdS* database; see *acdS* database supplemental material) representing the phylogenetic diversity of ACC deaminase producers defined based on published data [18–20, 50]. Dataset without singletons was used to generate rarefaction curves and Shannon, Simpson, and Chao diversity indices (calculated using sequencing subsample data for which each sample had the same number of sequences).

Alignment of selected *acdS* sequences (i.e., ten randomly chosen OTUs per genus) of the core-*acdS* database and eight related D-cystein desulfhydrase genes (see the “Identification of acdS primers” section) used as outgroup was carried out using MAFFT v7.123b (2013/10/15) [65, 66]. An *acdS* phylogenetic tree was constructed based on maximum likelihood method using RAxML 8.2.8 software [67]. Trees were annotated using iTOL V3 [68].

### Statistical analyses

Experimental treatments were compared based on log-numbers of *acdS* genes and mRNA, using ANOVA and Fisher’s LSD tests, in each of the soils and at each sampling time. Two-factor ANOVA and Fisher’s LSD tests were also done, to consider sampling time effects in cropped soil (9 treatment × 2 samplings) as well as past soil management at 21 days (4 treatments × 2 past soil managements). Comparisons for bacterial composition data were carried out by between-class analysis (BCA) (ADE4 R and ggplot2 packages) and Kruskal-Wallis rank sum test associated to Tukey’s HSD test.

To evaluate the influence of past *Poaceae* evolution on root interactions with *acdS* microorganisms, Spearman correlation analysis was carried out between the phylogenetic distance between *Poaceae* genotypes and the corresponding pairwise differences in (i) *acdS* gene or transcript raw numbers (based on Euclidean distances) or (ii) *acdS* microbial community composition (based on Bray-Curtis dissimilarity indices). The former was computed by Bouffaud et al. [36] from concatenated chloroplastic sequences of gene *rps16* and intergenic regions *rps16-trnK* and *atpI-atpH*, using the maximum likelihood method and Kimura two-parameter model.

All analyses were done at *P* < 0.05, using R 2.10.1 software (https://www.r-project.org).

### Nucleotide sequence accession numbers

Reads have been deposited in the European Bioinformatics Institute (EBI) database under accession number PRJEB24637.

## Additional files

Additional file 1: **Table S1.** Universal primers designed to target *acdS* alleles. **Table S2.** Universal primer pairs tested to amplify specifically *acdS* alleles, with selected primer pair indicated in bold. **Table S3.** Spearman correlation analysis of the relation of pairwise plant phylogenetic distance between *Poaceae* with various Euclidean distances between log-transformed qPCR data describing the corresponding *acdS* communities. (DOCX 74 kb)

Additional file 2: **Figure S1.** Examples of PCR amplification with the different primer pairs tested. (A) acdsF5/acdsR7; (B) acdsF5/acdsR8; (C) acdsF6/acdsR7; (D) acdsF6/acdsR8; (E) acdsF8/acdsR10. The different strains tested were 1. *Azospirillum lipoferum* 4B; 2. *A. lipoferum* TVV3; 3*. A lipoferum* CRT1 (*acdS*-); 4. *A. lipoferum* RSWT1; 5. *Burkholderia cepacia* LMG1222; 6. *B. cenocepacia* LMG16656; 7*. B. stabilis* LMG14294; 8. *B. dolosa* LMG18941; 9. *Pseudomonas thivervalensis* PITR2; 10. *P. kilonensis* F113; 11. *P. protegens* CHA0 (acdS-); and 12. *Ralstonia solanacearum* GMI1000. (TIFF 13453 kb)

Additional file 3: **Figure S2.** Example of the *acdS* amplification curves (A), standard curve (B), melting peaks (C) obtained using DNA from *Burkholderia cenocepacia* J2315, and (E) and (F) obtained using rhizospheric metagenomics DNA serially diluted. (TIF 602 kb)

Additional file 4: **Figure S3.** RAxML bipartition tree of 3322 sequenced *acdS* alleles from *Poaceae* rhizosphere. The tree was visualized using iTOL software. Branches colored in violet represent the out-group of D-cystein desulfhydrase genes, whereas *acdS* alleles affiliated to *Betaproteobacteria* are shown in khaki, to *Gammaproteobacteria* in blue, to *Actinobacteria* in green, to *Alphaproteobacteria* in red, and to microeukaryotes in orange. Branches are indicated in bold when corresponding to at least two genera (http://itol.embl.de/shared/acdStree). (PDF 892 kb)

Additional file 5: **Figure S4.** Rarefaction curves showing the number of microbial OTUs according to the number of *acdS* reads, based on observed data obtained from bulk soil or rhizosphere. Data for cropped soil are in full-lines and for meadow soil in dash-lines. (PDF 473 kb)

Additional file 6: **Figure S5.** Pairwise comparison of plant phylogenetic distance between *Poaceae* (*X*-axis) with the corresponding Euclidean distance between *acdS* reads for each of the 20 most abundant microbial genera representing over 90% of *acdS* functional group diversity (*Y*-axis). The 18 maize-based comparisons are indicated using black triangles (maize-maize; *n* = 6), diamonds (maize-teosinte; *n* = 4), circles (maize-sorghum; *n* = 4), or squares (maize-wheat; *n* = 4), and the three other comparisons using a white circle (teosinte-sorghum), white triangle (teosinte-wheat) or white square (sorghum-wheat). Distances were calculated two by two, using Kimura two-parameter model for plant phylogeny and Euclidean distance for taxa. (TIF 155 kb)

Additional file 7: **Figure S6.** Root (A), rhizosphere soil (B), and shoot (C) dry weights. Statistical analyses were performed independently at 21 days in cropped soil, at 21 days in meadow soil, and at 42 days in cropped soil, using ANOVA and Fisher LSD tests (*P* < 0.05; differences shown with letters a to d). For maize lines FV4, W85, tomato, and bulk soil, two-way ANOVA and Fisher LSD tests (*P* < 0.05) were also performed to compare treatments according to past soil management or sampling time, and differences with the same genotype at 21 days in cropped soil are indicated by symbols * and #, respectively. (PDF 115 kb)
