# Supplementary material for: 1-Aminocyclopropane-1-carboxylate deaminase producers associated to maize and other Poaceae species
Source: Microbiome. 2018 Jun 20;6:114. doi: 10.1186/s40168-018-0503-7 (PMC6011333; doi:10.1186/s40168-018-0503-7)
Supplement: Supplementary file 1 — Table S1. Universal primers designed to target acdS alleles. Table S2. Universal primer pairs tested to amplify specifically acdS alleles, with selected primer pair indicated in bold. Table S3. Spearman correlation analysis of the relation of pairwise plant phylogenetic distance between Poaceae with various Euclidean distances between log-transformed qPCR data describing the corresponding acdS communities. (DOCX 74 kb) [file 40168_2018_503_MOESM1_ESM.docx]

| **Table S1**. Universal primers designed to target *acdS* alleles | | | | |
| --- | --- | --- | --- | --- |
| Name | Primer sequence 5' → 3' | Size (bp) | Tm (°C) | Position in *acdS* of *Burkholderia cenocepacia* LMG 16656 |
| acdSF1 | CCSACRCCSATCGAGMAGCT | 20 | 65 | 43 |
| acdSF2 | CCRCACGAGGAYGCSGTCT | 19 | 77.5 | 310 |
| acdSR2 | AGACSGCRTCCTCGTGYGG | 19 | 77.5 | 308 |
| acdSF3 | AGKMGGTVAAGGMYGCSGGC | 20 | 77.4 | 437 |
| acdSR3 | GCCSGCRKCCTTBACCKMCT | 20 | 77.4 | 437 |
| acdSR4 | CGSGCSGMSAGVCGGAT | 17 | 82.8 | 835 |
| acdSR4bis | GCSMSAGVCGGATSGCCT | 18 | 81.7 | 830 |
| acdSF5 | GGCAACAAGMYSCGCAAGCT | 20 | 76.6 | 145 |
| acdSF6 | GCSAAGCGCGASGACTGCAA | 20 | 80.8 | 106 |
| acdSF7 | CAGTCSAACCASACSCGC | 18 | 72.7 | 229 |
| acdSR7 | GCGSGTSTGGTTSGACTG | 18 | 72.8 | 229 |
| acdSF8 | TGAAGTGCGTSCTSGTGCAG | 20 | 75.7 | 278 |
| acdSR8 | CTGCACSAGSACGCACTTCA | 20 | 75.7 | 278 |
| acdSF9 | CAGGARGTGCGSSMGCAGGA | 20 | 81.9 | 529 |
| acdSR9 | TCCTGCKSSCGCACYTCCTG | 20 | 81.9 | 529 |
| acdSF10 | GTSTGCTCGGTSACSGGC | 18 | 75 | 565 |
| acdSR10 | GCCSGTSACCGAGCASAC | 18 | 75 | 583 |

| **Table S2**. Universal primers pairs tested to amplify specifically *acdS* alleles, with selected primer pair indicated in bold | | | | | |  |
| --- | --- | --- | --- | --- | --- | --- |
| Primer pair | Product length (bp) | PCR amplification problems on tested strains* | | | |  |
| acdSF1 + acdSR2 | 283 | Not specific |  |  |  |  |
| acdSF2 + acdSR3 | 147 | Not universal (3 tested strains never amplified) | | | |  |
| acdSF3 + acdSR4 | 415 | False positive |  |  |  |  |
| acdSF3 + acdSR4bis | 411 | False positive |  |  |  |  |
| acdSF5 + acdSR7 | 102 | False positive |  |  |  |  |
| **acdSF5 + acdSR8** | **153** | **-** |  |  |  |  |
| acdSF6 + acdSR7 | 143 | Not universal (3 tested strains never amplified) | | | |  |
| acdSF6 + acdSR8 | 192 | Not specific | | |  |  |
| acdSF7 + acdSR10 | 372 | Not universal (5 tested *acdS*+ strain never amplified) | | | |  |
| acdSF7 + acdSR9 | 320 | False positive |  |  |  |  |
| acdSF8 + acdSR10 | 323 | False positive |  |  |  |  |
| acdSF8 + acdSR10 | 323 | False positive |  |  |  |  |
| acdSF8 + acdSR9 | 271 | False positive |  |  |  |  |
| *tested strains were *Azospirillum lipoferum* TVV3, *A. lipoferum* 4B, *A. lipoferum* RSWT1, *Burkholderia cepacia* LMG 1222, *B. cenocepacia* LMG 16656, *B. cenocepacia* J2315*, B. stabilis* LMG 14294, *B. dolosa* LMG18941, *Pseudomonas thivervalensis* PITR2, *P. kilonensis* F113, *Ralstonia solanacearum* GMI1000 and the non-*acdS* strains *P. protegens* CHA0, *Escherichia coli* K12 and *A. lipoferum* CRT1. | | | | | | |

| **Table S3**. Spearman correlation analysis of the relation of pairwise plant phylogenetic distance between *Poaceae* with various Euclidean distances between log-transformed qPCR data describing the corresponding *acdS* communities | | | | | |
| --- | --- | --- | --- | --- | --- |
|  | *acdS* genes per g soil | *acdS* transcripts per g soil | *acdS* genes per g root | *acdS* transcripts per g root |  |
| *P* value | 0.78 | 0.08 | 0.84 | 0.19 |  |
| Rho | 0.07 | 0.40 | 0.05 | -0.13 |  |
