# Supplementary figures and images for: 1-Aminocyclopropane-1-carboxylate deaminase producers associated to maize and other Poaceae species
Source: Microbiome. 2018 Jun 20;6:114. doi: 10.1186/s40168-018-0503-7 (PMC6011333; doi:10.1186/s40168-018-0503-7)

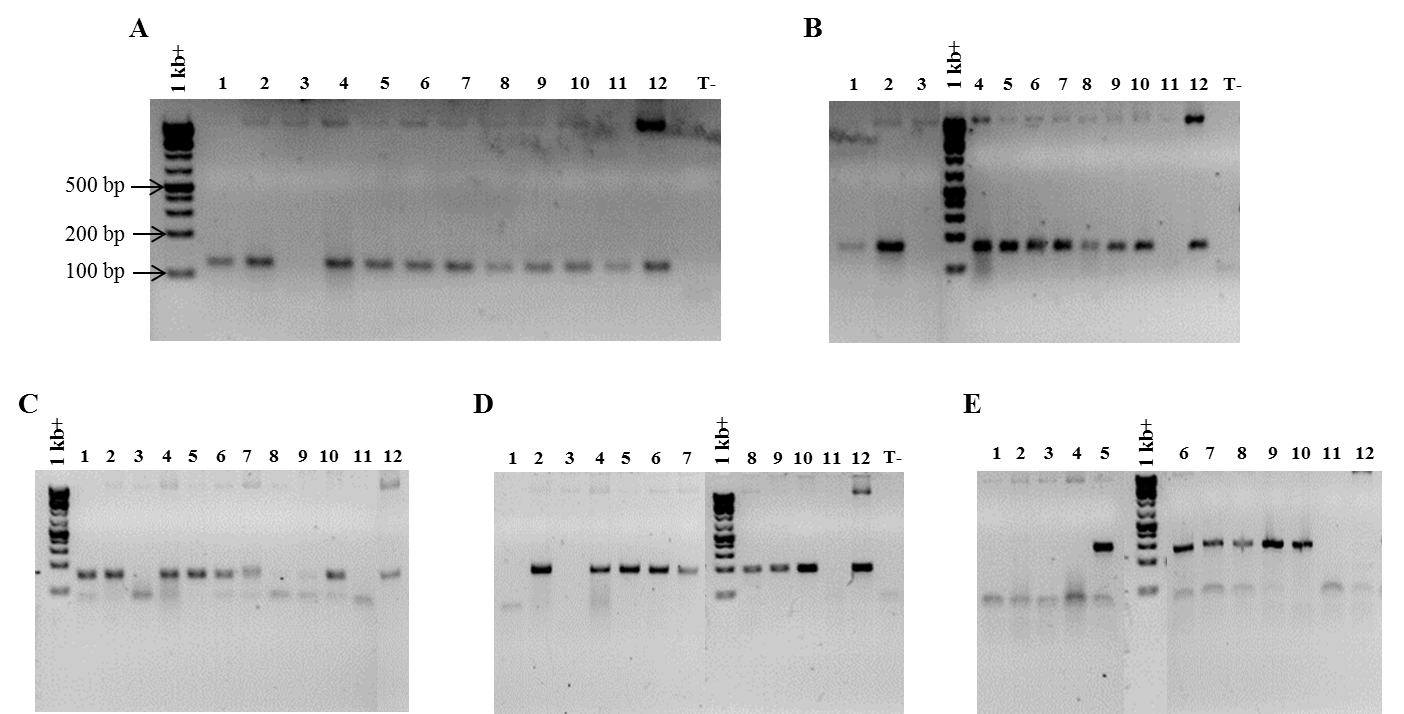

Supplement: Supplementary file 2 — Figure S1. Examples of PCR amplification with the different primer pairs tested. (A) acdsF5/acdsR7; (B) acdsF5/acdsR8; (C) acdsF6/acdsR7; (D) acdsF6/acdsR8; (E) acdsF8/acdsR10. The different strains tested were 1. Azospirillum lipoferum 4B; 2. A. lipoferum TVV3; 3. A lipoferum CRT1 (acdS-); 4. A. lipoferum RSWT1; 5. Burkholderia cepacia LMG1222; 6. B. cenocepacia LMG16656; 7. B. stabilis LMG14294; 8. B. dolosa LMG18941; 9. Pseudomonas thivervalensis PITR2; 10. P. kilonensis F113; 11. P. protegens CHA0 (acdS-); and 12. Ralstonia solanacearum GMI1000. (TIFF 13453 kb) [file 40168_2018_503_MOESM2_ESM.tiff]

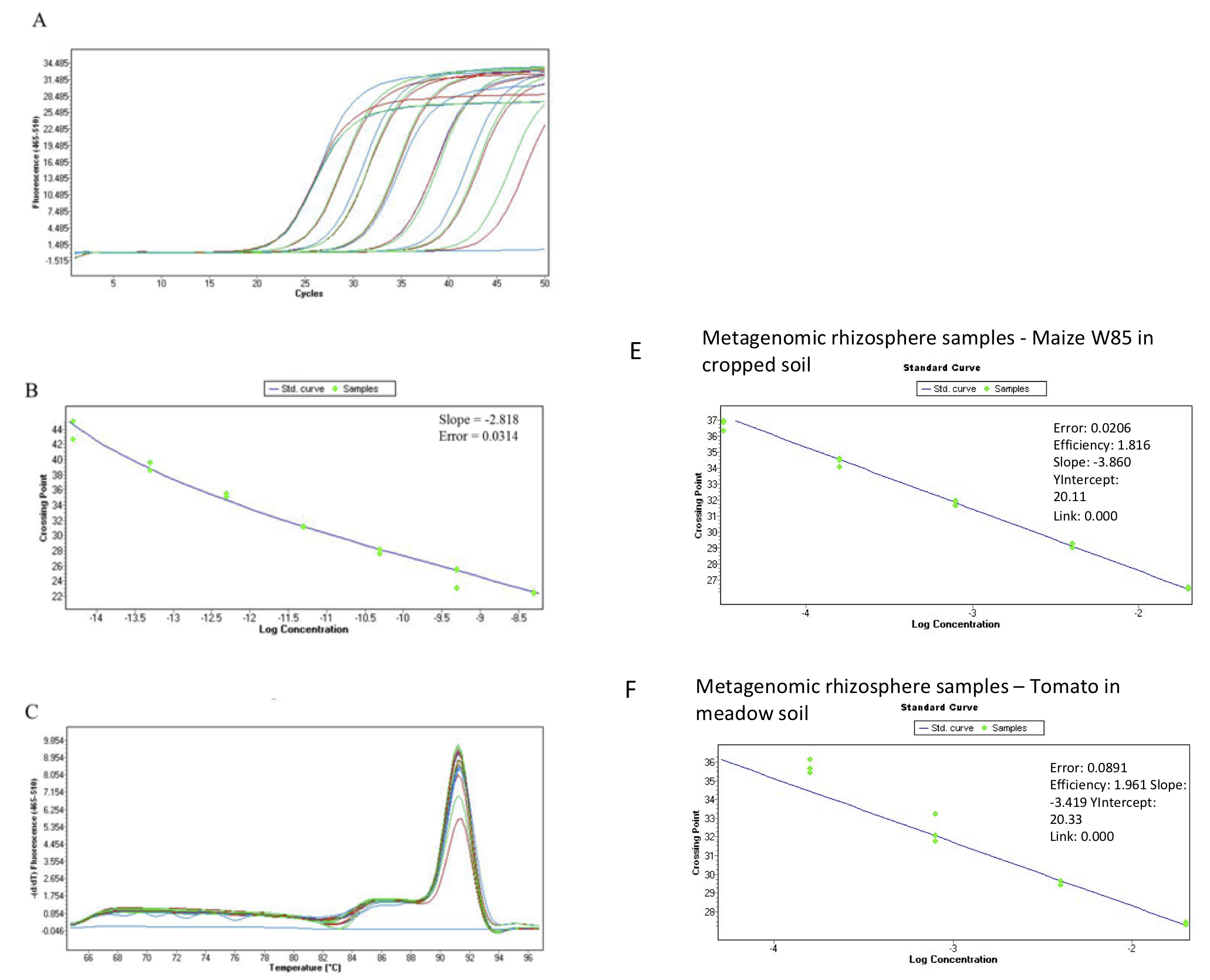

Supplement: Supplementary file 3 — Figure S2. Example of the acdS amplification curves (A), standard curve (B), melting peaks (C) obtained using DNA from Burkholderia cenocepacia J2315, and (E) and (F) obtained using rhizospheric metagenomics DNA serially diluted. (TIF 602 kb) [file 40168_2018_503_MOESM3_ESM.tif]

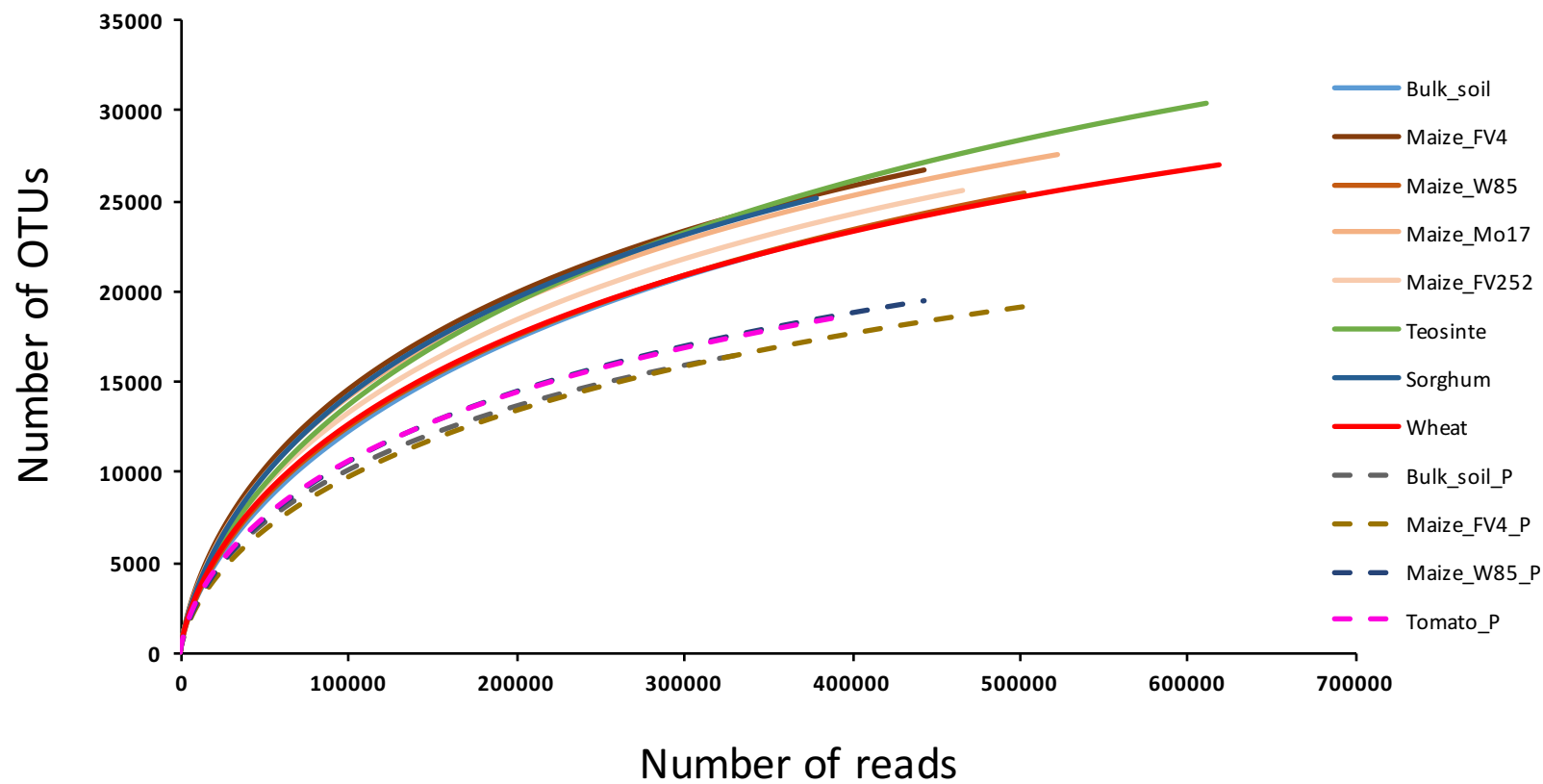

Figure S2

Supplement: Supplementary file 5 — Figure S4. Rarefaction curves showing the number of microbial OTUs according to the number of acdS reads, based on observed data obtained from bulk soil or rhizosphere. Data for cropped soil are in full-lines and for meadow soil in dash-lines. (PDF 473 kb) [file 40168_2018_503_MOESM5_ESM.pdf]

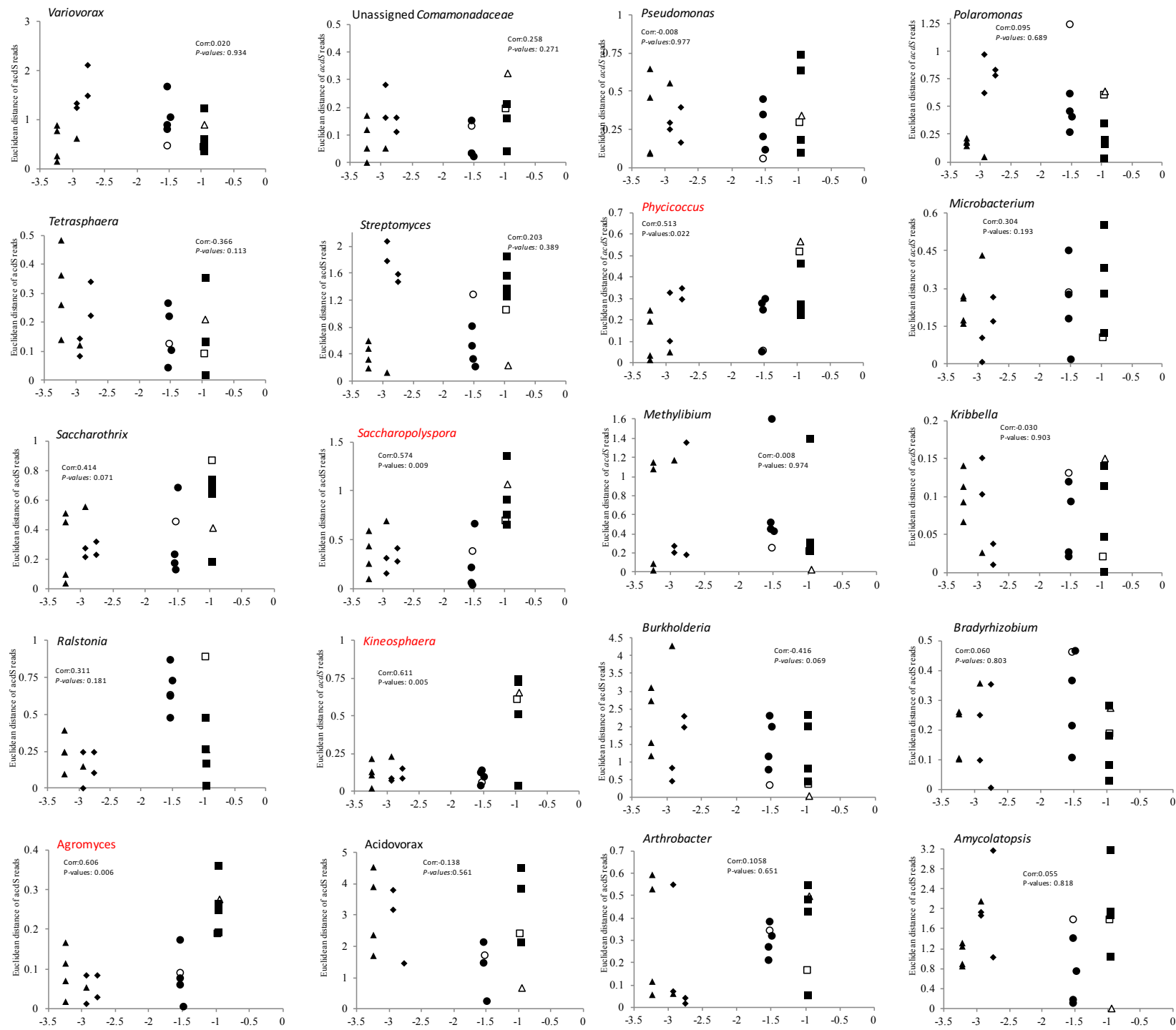

Figure S3

Supplement: Supplementary file 6 — Figure S5. Pairwise comparison of plant phylogenetic distance between Poaceae (X-axis) with the corresponding Euclidean distance between acdS reads for each of the 20 most abundant microbial genera representing over 90% of acdS functional group diversity (Y-axis). The 18 maize-based comparisons are indicated using black triangles (maize-maize; n = 6), diamonds (maize-teosinte; n = 4), circles (maize-sorghum; n = 4), or squares (maize-wheat; n = 4), and the three other comparisons using a white circle (teosinte-sorghum), white triangle (teosinte-wheat) or white square (sorghum-wheat). Distances were calculated two by two, using Kimura two-parameter model for plant phylogeny and Euclidean distance for taxa. (TIF 155 kb) [file 40168_2018_503_MOESM6_ESM.tif]

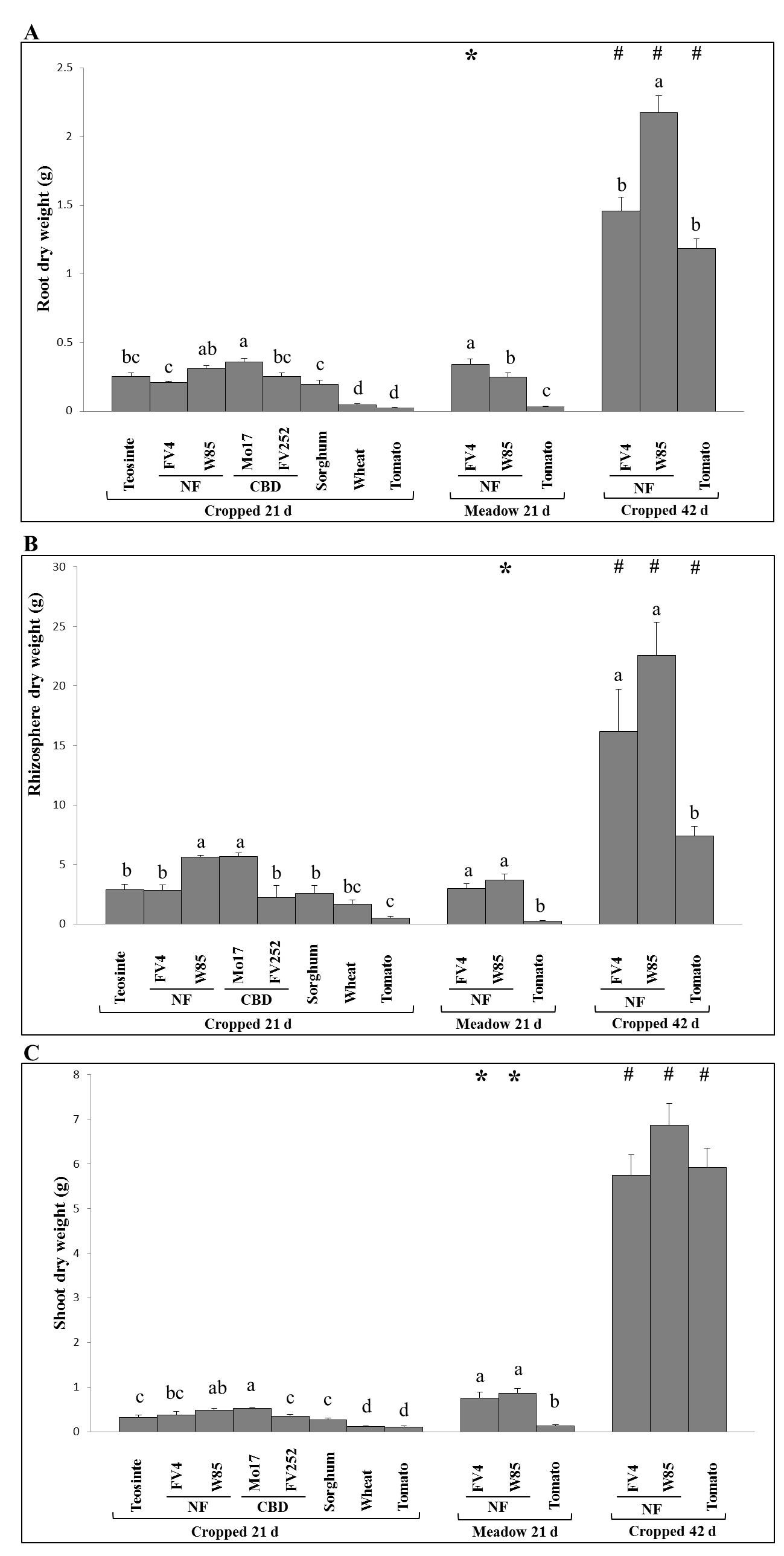

Supplement: Supplementary file 7 — Figure S6. Root (A), rhizosphere soil (B), and shoot (C) dry weights. Statistical analyses were performed independently at 21 days in cropped soil, at 21 days in meadow soil, and at 42 days in cropped soil, using ANOVA and Fisher LSD tests (P < 0.05; differences shown with letters a to d). For maize lines FV4, W85, tomato, and bulk soil, two-way ANOVA and Fisher LSD tests (P < 0.05) were also performed to compare treatments according to past soil management or sampling time, and differences with the same genotype at 21 days in cropped soil are indicated by symbols * and #, respectively. (PDF 115 kb) [file 40168_2018_503_MOESM7_ESM.pdf]
