# Supplementary material for: 1-Aminocyclopropane-1-carboxylate deaminase producers associated to maize and other Poaceae species
Source: Microbiome. 2018 Jun 20;6:114. doi: 10.1186/s40168-018-0503-7 (PMC6011333; doi:10.1186/s40168-018-0503-7)
Supplement: Supplementary file 4 — Figure S3. RAxML bipartition tree of 3322 sequenced acdS alleles from Poaceae rhizosphere. The tree was visualized using iTOL software. Branches colored in violet represent the out-group of D-cystein desulfhydrase genes, whereas acdS alleles affiliated to Betaproteobacteria are shown in khaki, to Gammaproteobacteria in blue, to Actinobacteria in green, to Alphaproteobacteria in red, and to microeukaryotes in orange. Branches are indicated in bold when corresponding to at least two genera (http://itol.embl.de/shared/acdStree). (PDF 892 kb) [file 40168_2018_503_MOESM4_ESM.pdf]

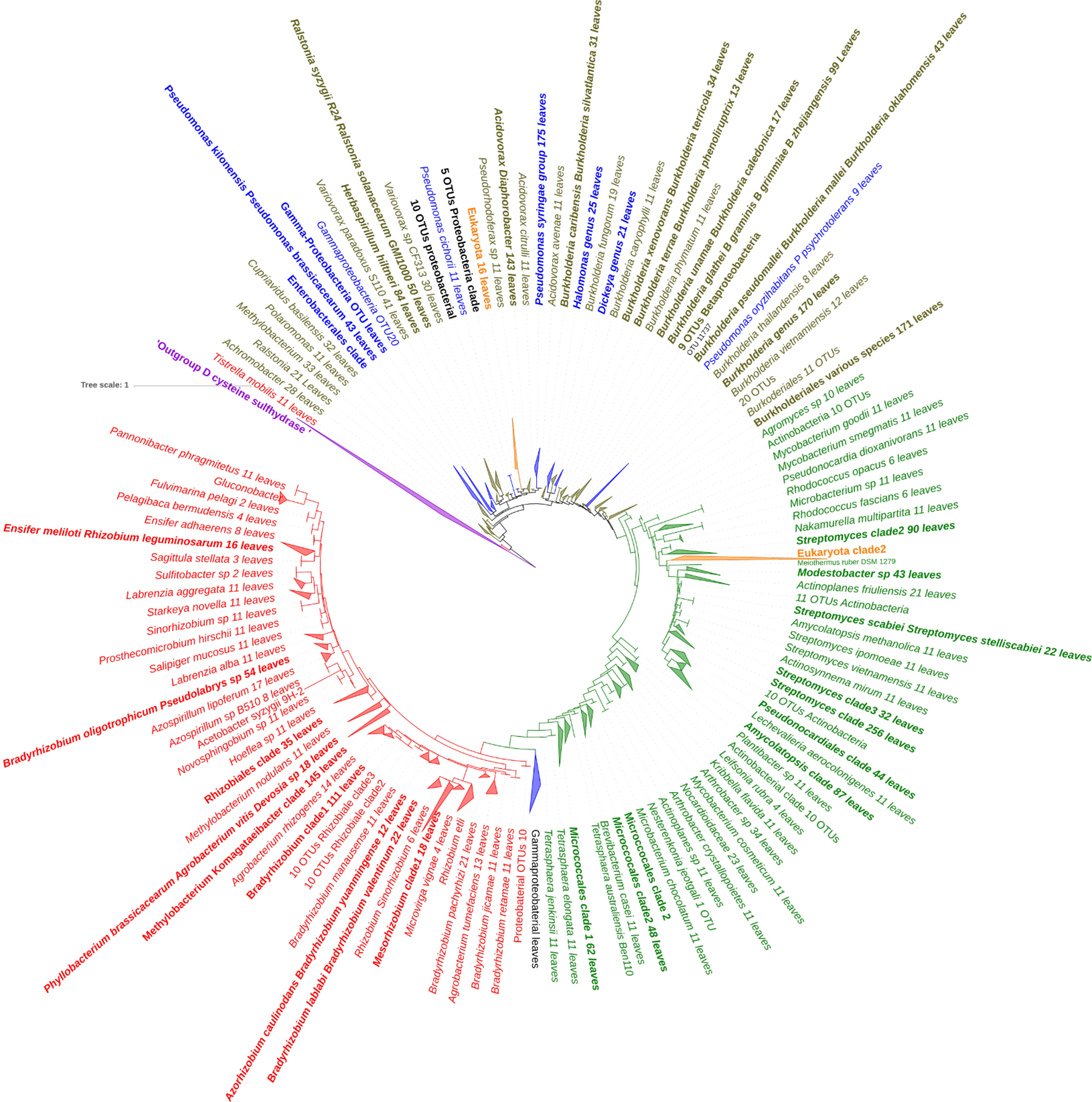

Tree scale: 1
'Outgroup D cysteine sulfhydrase'
Tistrella mobilis 11 leaves
Achromobacter 28 leaves
Ralstonia 21 Leaves
Methylobacterium 33 leaves
Polaromonas 11 leaves
Cupriavidus basilensis 32 leaves
Enterobacterales clade
Gamma-Proteobacteria OTU leaves
Pseudomonas kilonensis Pseudomonas brassicacearum 43 leaves
Gammaproteobacteria OTU20
Variovorax paradoxus S110 41 leaves
Herbaspirillum hiltneri 84 leaves
Ralstonia syzygii R24 Ralstonia solanacearum GMI1000 50 leaves
Variovorax sp CF313 30 leaves
10 OTUs proteobacterial
Pseudomonas cichorii 11 leaves
5 OTUs Proteobacteria clade
Eukaryota 16 leaves
Pseudorhodoferax sp 11 leaves
Acidovorax Diaphorobacter 143 leaves
Acidovorax citrulli 11 leaves
Pseudomonas syringae group 175 leaves
Acidovorax avenae 11 leaves
Burkholderia caribensis Burkholderia silvatlantica 31 leaves
Halomonas genus 25 leaves
Burkholderia fungorum 19 leaves
Dickeya genus 21 leaves
Burkholderia caryophylli 11 leaves
Burkholderia xenovorans Burkholderia terricola 34 leaves
Burkholderia terrae Burkholderia phenoliruptrix 13 leaves
Burkholderia phymatum 11 leaves
Burkholderia unamae Burkholderia caledonica 17 leaves
Burkholderia glathei B graminis B grimmiae B zhejiangensis 99 Leaves
9 OTUs Betaproteobacteria
OTU 11737
Burkholderia pseudomallei Burkholderia mallei Burkholderia oklahomensis 43 leaves
Pseudomonas oryzihabitans P psychrotolerans 9 leaves
Burkholderia thailandensis 8 leaves
Burkholderia genus 170 leaves
Burkholderia vietnamiensis 12 leaves
Burkholderia 11 OTUs
20 OTUs
Burkholderia various species 171 leaves
Burkoderiales 11 OTUs
Agromyces sp 10 leaves
Actinobacteria 10 OTUs
Mycobacterium goodii 11 leaves
Mycobacterium smegmatis 11 leaves
Pseudonocardia dioxanivorans 11 leaves
Rhodococcus opacus 6 leaves
Microbacterium sp 11 leaves
Rhodococcus fascians 6 leaves
Nakamurella multipartita 11 leaves
Streptomyces clade2 90 leaves
Eukaryota clade2
Meiothermus ruber DSM 1279
Modestobacter sp 43 leaves
Actinoplanes friuliensis 21 leaves
11 OTUs Actinobacteria
Streptomyces scabiei Streptomyces stelliscabiei 22 leaves
Amycolatopsis methanolica 11 leaves
Streptomyces ipomoeae 11 leaves
Streptomyces vietnamensis 11 leaves
Actinosynnema mirum 11 leaves
Streptomyces clade3 32 leaves
Streptomyces clade 256 leaves
10 OTUs Actinobacteria
Pseudonocardiales clade 44 leaves
Lechevalieria aerocolonigenes 11 leaves
Amycolatopsis clade 87 leaves
Plantibacter sp 11 leaves
Actinobacterial clade 10 OTUs
Leifsonia rubra 4 leaves
Kribbella flavida 11 leaves
Arthrobacter sp 34 leaves
Mycobacterium cosmeticum 11 leaves
Nocardioidaceae 23 leaves
Arthrobacter crystallopoietes 11 leaves
Actinoplanes sp 11 leaves
Nesterenkonia jeotgali 1 OTU
Microbacterium chocolatum 11 leaves
Micrococcales clade2 48 leaves
Micrococcales clade 2
Brevibacterium casei 11 leaves
Tetrasphaera australiensis Ben110
Micrococcales clade 1 62 leaves
Tetrasphaera elongata 11 leaves
Tetrasphaera jenkinsii 11 leaves
Gammaproteobaterial leaves
Proteobaterial OTUs 10
Bradyrhizobium retamae 11 leaves
Bradyrhizobium jicamae 11 leaves
Agrobacterium tumefaciens 13 leaves
Bradyrhizobium pachyrhizi 21 leaves
Rhizobium etli
Microvirga vignae 4 leaves
Mesorhizobium clade1 18 leaves
Rhizobium Sinorhizobium 6 leaves
Bradyrhizobium yuanmingense 12 leaves
Bradyrhizobium lablabi Bradyrhizobium valentinum 22 leaves
Azorhizobium caulinodans
Bradyrhizobium manausense 11 leaves
10 OTUs Rhizobiale clade2
10 OTUs Rhizobiale clade3
Bradyrhizobium clade1 111 leaves
Agrobacterium rhizogenes 14 leaves
Methylobacterium Komagataeibacter clade 145 leaves
Phyllobacterium brassicacearum Agrobacterium vitis Devosia sp 18 leaves
Methylobacterium nodulans 11 leaves
Rhizobiales clade 35 leaves
Hoeflea sp 11 leaves
Novosphingobium sp 11 leaves
Acetobacter syzygii 9H-2
Azospirillum sp B510 8 leaves
Azospirillum lipoferum 17 leaves
Bradyrhizobium oligotrophicum Pseudolabrys sp 54 leaves
Labrenzia alba 11 leaves
Salipiger mucosus 11 leaves
Prosthecomicrobium hirschii 11 leaves
Sinorhizobium sp 11 leaves
Starkeya novella 11 leaves
Labrenzia aggregata 11 leaves
Sulfitobacter sp 2 leaves
Sagittula stellata 3 leaves
Ensifer meliloti Rhizobium leguminosarum 16 leaves
Ensifer adhaerens 8 leaves
Pelagibaca bermudensis 4 leaves
Fulvimarina pelagi 2 leaves
Gluconobacter
Pannonibacter phragmitetus 11 leaves

Figure S1
